# Military Service and National Health

**Published:** 1900-02-24

**Authors:** 


### The Hospital

A Journal of

The Medical Sciences and Hospital Administration.

Vol. XXVII.—No. 700.] Edited by Sir Henry Burdett, K.C.B., and Solomon C. Smith, M.D., M.R.C.P. [February 24, 1900.

### Military Service and National Health.

It would hardly be possible for any observer of current events to lose sight of their manifest tendency towards an enlargement of the military forces of the Empire. The word "conscription" has made its appearance in more than one authoritative organ of public opinion, and the restoration of a general ballot for the militia has been advocated in many quarters. Such a procedure would only become necessary if the number of militiamen called for by the Queen in Council from any given locality were not furnished by voluntary enlistment. Moreover, a mere augmentation of the militia alone could only meet the necessities of the case by liberating an equivalent number of regulars for service wherever they might be required. It is conceivable that such a course might dangerously denude our own shores of trained defenders, in which case it might become advisable to provide for a considerable augmentation of the regular forces. This might obviously be done by pecuniary or other inducements to men of the proper class to enlist for long periods, and so to make the Army their definite and settled calling; or it might be done by casting the net of short service over a much larger number of persons than are now caught within its meshes. The matter, as one of public economy, requires to be considered from various points of view, financial, moral, industrial, and with reference to emigration; while it can hardly be denied that, in some of its aspects, it may come to have an important bearing upon questions of public health. It is under this aspect that we propose to consider it, leaving all other advantages or disadvantages to be duly weighed by those who will be called upon to accept responsibility for any recommendations which they may feel called upon to submit to Parliament.

The system of conscription was introduced into France rather more than 100 years ago, by an ordinance which declared every citizen to be liable to military service for the five years between the ages of twenty and twenty-five, and which provided that everyone should be enrolled by name and called upon if he were wanted. With some modifications the same principle has been applied in most European countries; but in Prussia, as a consequence of the limitation in the numbers of the army fixed by the treaty of Tilsit, a term of shorter service was introduced, by which the Government was enabled to pass a larger number of men through the ranks in the same time, and to disperse them again among the civil population ready to be called back to the colours in case of need. Much has been said about the industrial dislocation thus occasioned; and it has been zealously maintained by some, and as zealously denied by others, that the improved habits insensibly acquired under the influence of military discipline, and carried back by the discharged soldier into his ordinary duties, more than compensate, in the increased efficiency which they confer, for the period of time which may have been occupied in acquiring them. It seems difficult, and would perhaps be impossible, to arrive at any accurate common measure by which the time and the results could be compared; and the controversy may perhaps be held to establish that there is a fairly even balance between the two. With regard to the probable effects of general military service upon public health, we are inclined to think that it would be altogether beneficial. The aggregation of units rendered necessary by military life has in former times occasioned barracks to be hotbeds of disease; but these same hotbeds have yielded the incidental advantages of enabling the medical profession to demonstrate the causes of such disease by experiments on the largest scale, and to show by what means they might be effectually removed. The average annual mortality of the Foot Guards between the years 1826 and 1846 was 20·5 per thousand, or more than double that of the total male population within the same limits of age, and this excess was chiefly due to the insanitary state of barracks and guard-rooms. In the year 1884 the mortality among the Foot Guards was 8·27 per thousand, or 1·96 per thousand below that of all males of corresponding ages, but was still 2·94 higher than that of all arms in the United Kingdom for the same year. It is unfortunate that the invaluable supplement to the fifty-fifth report of the Registrar-General, containing Dr. Tatham's conclusions on occupational mortality, contains no reference to soldiers as such; but there can be no doubt either of the general healthiness of the army, or of the immense improvement in physique which is one of the earliest consequences of drill. The erect carriage and the expanded chest provide for a completeness of aeration of the blood to which the average young man of the private soldier class is an absolute stranger; the food is better and more nutritious than any he would obtain at home; and the compulsory cleanliness which is one of the primary requirements of the army, and which can scarcely fail to become habitual when once the comfort incidental to it has been experienced, is, we need hardly say, a powerful protection against disease.

